# Supplementary material for: Developmental pruning of sensory neurites by mechanical tearing in Drosophila
Source: J Cell Biol. 2023 Jan 17;222(3):e202205004. doi: 10.1083/jcb.202205004 (PMC9856751; doi:10.1083/jcb.202205004)

from high resolution tiff files

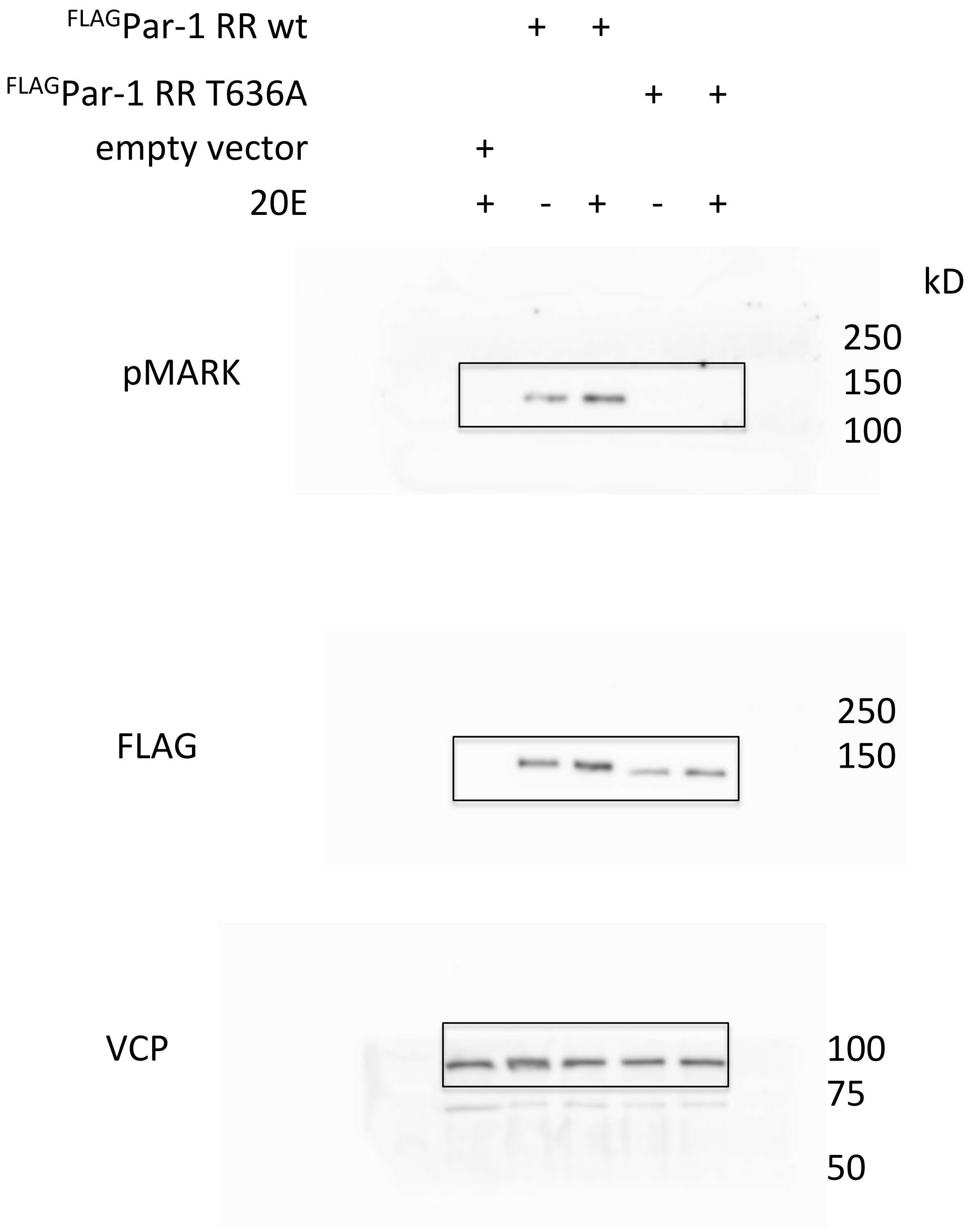

with molecular weight markers, lower resolution

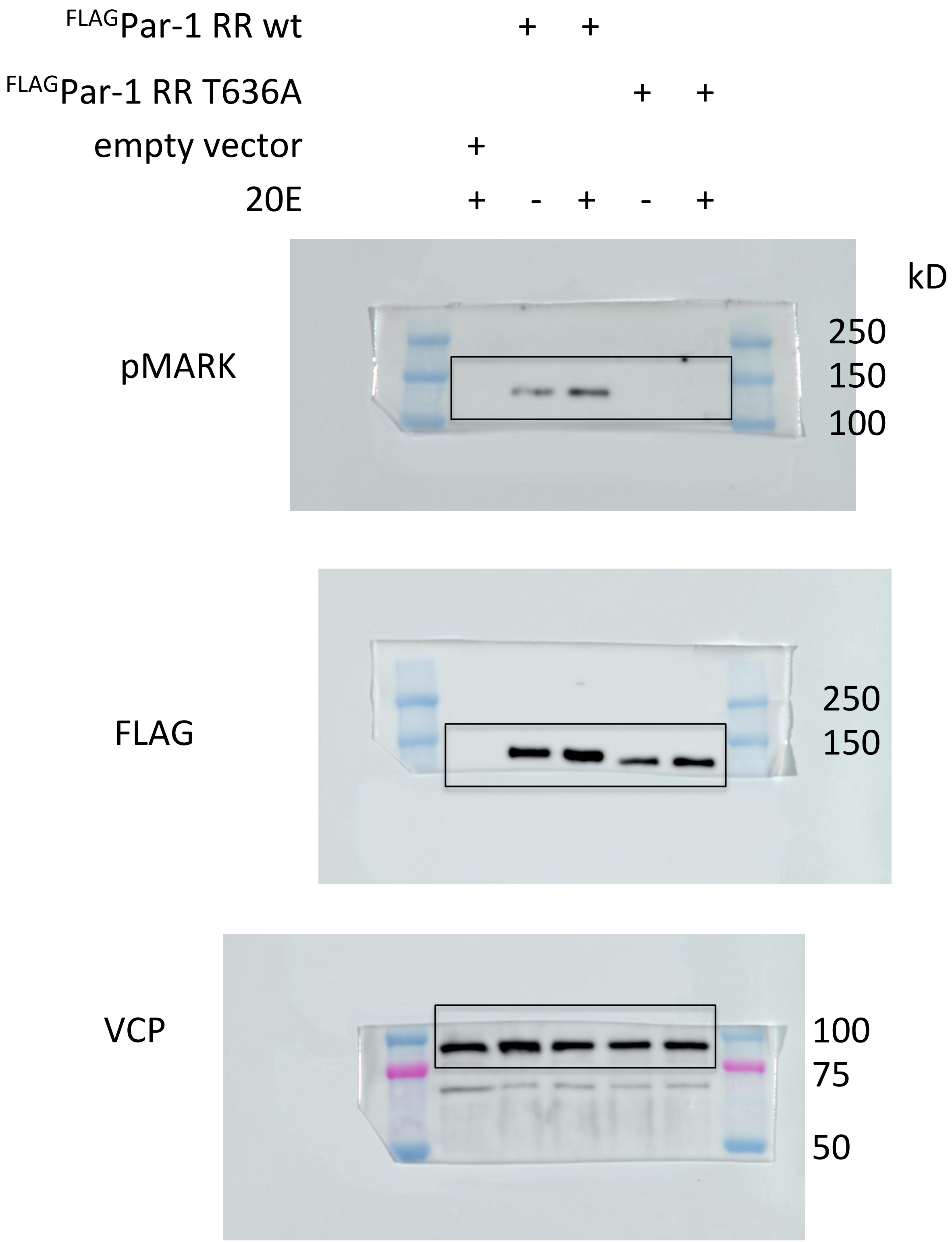

Supplement: SourceData FS1 — is the source file for Fig. S1. [file JCB_202205004_SourceDataFS1.pdf]
